# Grape Pomace for Topical Application: Green NaDES Sustainable Extraction, Skin Permeation Studies, Antioxidant and Anti-Inflammatory Activities Characterization in 3D Human Keratinocytes

**DOI:** 10.3390/biom11081181

**Published:** 2021-08-10

**Authors:** Angela Punzo, Emanuele Porru, Alessia Silla, Patrizia Simoni, Paola Galletti, Aldo Roda, Emilio Tagliavini, Chiara Samorì, Cristiana Caliceti

**Affiliations:** 1 Department of Chemistry “Giacomo Ciamician”, Alma Mater Studiorum-University of Bologna, 40126 Bologna, Italy; angela.punzo2@unibo.it (A.P.); emanuele.porru2@unibo.it (E.P.); paola.galletti@unibo.it (P.G.); aldo.roda@unibo.it (A.R.); emilio.tagliavini@unibo.it (E.T.); chiara.samori@unibo.it (C.S.); 2 Department of Biomedical and Neuromotor Sciences, Alma Mater Studiorum-University of Bologna, 40126 Bologna, Italy; alessia.silla2@unibo.it; 3 Department of Medical and Surgical Sciences, Alma Mater Studiorum-University of Bologna, 40138 Bologna, Italy; patrizia.simoni@unibo.it; 4 Interdepartmental Centre for Renewable Sources, Environment, Sea and Energy (CIRI FRAME), Alma Mater Studiorum-University of Bologna, 48123 Bologna, Italy; 5 Biostructures and Biosystems National Institute (INBB), 00136 Rome, Italy

**Keywords:** red grape pomace skin, natural deep eutectic solvents (NaDES), malvidin, skin permeation, human 3D keratinocytes, oxidative stress, inflammation

## Abstract

Food waste is a global problem due to its environmental and economic impact, so there is great demand for the exploitation of new functional applications. The winemaking process leads to an incomplete extraction of high-value compounds, leaving the pomace still rich in polyphenols. This study was aimed at optimising and validating sustainable routes toward the extraction and further valorisation of these polyphenols, particularly for cosmeceutical applications. New formulations based on red grape pomace polyphenols and natural deep eutectic solvents (NaDESs) were here investigated, namely betaine combined with citric acid (BET-CA), urea (BET-U) and ethylene glycol (BET-EG), in which DESs were used both as extracting and carrying agents for polyphenols. The flavonoid profile determined by HPLC-MS/MS analysis showed similar malvidin content (51–56 μg mL^−1^) in the DES combinations, while BET-CA gave the best permeation performance in Franz cells, so it was further investigated in 3D human keratinocytes (HaCat spheroids) injured with the pro-oxidant agent menadione. BET-CA treatment showed good intracellular antioxidant activity (IC50 0.15 ± 0.02 μg mL^−1^ in malvidin content) and significantly decreased (*p* < 0.001) the release of the pro-inflammatory cytokine IL-8, improving cell viability. Thus, BET-CA formulation is worthy of investigation for potential use as a cosmetic ingredient to reduce oxidative stress and inflammation, which are causes of skin aging.

## 1. Introduction

Each year, an estimated one-third of all food produced—equivalent to 1.3 billion tonnes worth around USD 1 trillion—ends up rotting in the bins of consumers and retailers or spoiling due to poor transportation and harvesting practices. Food waste is also a serious problem for food processing industries, especially when it represents a loss of a valuable source of nutrients and phytochemicals [1]. Exploitation of new extraction processes and further valorisation of these by-products can increase resource efficiency and promote sustainable lifestyles.

Italy is a traditional wine-producing country and one of the world’s leading wine producers, ranked first for volume of exports and second after France for value [2]. During winemaking, only a minor part of the grape’s phytochemicals are extracted into the wine, so grape pomace, representing about 20%wt of the processed grapes, is enriched in high-value compounds, such as polyphenols [3].

Regulation EC 479\2008 of the European Union introduced important new elements to the legal framework of the wine sector, offering member states the possibility of defining the end-use of pomace. Italy implemented the above regulation through a ministerial decree, which, as an alternative to distillation, allows it to be collected under control for the recovery of anthocyanins, the production of agricultural products (direct or indirect agronomical use), energy recovery (using by-products such as biomass for the production of biogas or for fuelling energy-production plants) and the extraction of molecules with a high added-value for pharmaceutical and cosmetic purposes.

When the target compounds of grape pomace are soluble or weakly bound, such as polyphenols, the most common extraction technique is solid–liquid extraction, mainly based on toxic organic solvents [4].

Here, we exploited the profiles of the major classes of flavonoids present in red grape skin of traditional varieties of the Emilia Romagna region (“Sangiovese” red wines), typically cultivated in a niche area in northern Italy, along with their bioavailability and biological activities for innovative cosmeceutical applications. Specifically, we developed novel eco-friendly formulations based on polyphenols extracted with natural deep eutectic solvents (NaDESs), aiming at demonstrating a novel approach for the valorisation of winery by-products for human skin protection. NaDESs are playing an increasingly relevant role in the field of the extraction of polar molecules (like polyphenols) from natural matrices [5]; however, the use of NaDESs as polyphenols carriers, incorporating NaDESs into bioactive formulations, is a relatively new concept [6]. This is mainly because the exploitation of NaDESs as a part of bioactive formulations for peculiar biological applications, like cosmetic ones, requires a strict evaluation of their potential toxic effects (in the present case against skin cells).

The NaDESs used in the present study were based on betaine and three different hydrogen bond donors (urea, citric acid and ethylene glycol), chosen based on their proven biocompatibility.

The low harmfulness/toxicity and the compatibility of these single components are well-established and their use in the cosmetic field is legally allowed; moreover, the characterization of NaDES extracts used in this work in sugars and total polyphenols content has already been performed [4]. However, a deep study in anthocyanin and flavonol profiles has not been detailed so far and it is still not clear if there is a correlation between the composition of NaDES extracts and their biological effects in human skin. To this purpose, keratinocytes were used here as a cell model to directly verify the suitability of polyphenol–NaDES formulations in the cosmetic field. Keratinocytes play a major role in the structural and barrier functions of the epidermis [7], so they have been regarded as a prime choice for non-animal skin toxicity assessment to evaluate grape pomace bioactivity [8,9]. However, two-dimensional (2D) human keratinocytes have several limitations when they are used as a substitute for animal experiments. The main limitations include their failure to replicate the in vivo complexity and actual cellular architecture comprising the extracellular matrix (ECM) microenvironment and cell–cell interactions. Three-dimensional cell culture systems overcome many of the limitations of traditional 2D cell culture systems, more closely mimicking the complex phenotypic heterogeneity that chemical gradients produce during cell growth. In the current study, 3D keratinocytes were thus used to investigate the potential beneficial skin activities of grape pomace extracts [10].

The innovative extraction with NaDESs combined with a validated bioanalytical approach—based on the assessment of anthocyanin content in pomace extracts and their bioavailability in the skin in NaDES formulations, along with their relationship with in vitro beneficial activity in human 3D keratinocytes—represents a smart and powerful tool for complete cosmeceutical profiling. Our final aim was to propose a correct and sustainable approach to better reutilizing pomace extracts in order to open new perspectives on their use for possible future applications in the cosmetic field.

## 2. Materials and Methods

### 2.1. Chemicals

The chemiluminescent probe (AquaSpark™ 510 Peroxide Probe) was provided by Biosynth Carbosynth (Staad, Switzerland). A 10 mM stock solution was prepared by solubilizing the probe in DMSO. This solution is stable for months when stored at 4 °C in the dark. Phosphate-buffered saline (PBS) tabs (giving a 137 mM NaCl, 2.7 mM KCl phosphate buffer solution, pH 7.4, final concentration 0.01M), H_2_O_2_ solution, trypsin-EDTA, oxidative stress inductor 2-methyl-1,4-naphthoquinone (menadione), malvidin, the antibiotic solution 100× (10,000 U mL^−1^ penicillin and 10 mg mL^−1^ streptomycin) and the internal standard hydroxy-tyrosol for HPLC-ESI-MS/MS analysis were purchased from Sigma-Aldrich (St Louis, MO, USA). Anthocyanins standards for HPLC-ESI-MS/MS analysis were bought from Biosynth Carbosynth (Staad SG, SANKT GALLEN, Switzerland). A stock solution of menadione (100 mM) was prepared in DMSO. Dulbecco’s Modified Eagle Medium (DMEM) high glucose was purchased from Microgem (Naples, Italy) and fetal bovine serum (FBS) was purchased from Thermo Fischer Scientific (Waltham, MA, USA). The lactate dehydrogenase (LDH) cytotoxicity assay kit was purchased from Dojindo Molecular Technologies (Rockville, MD, USA).

### 2.2. Red Pomace Materials and Extracts

Red grape pomace from red grape (*Vitis vinifera* L.) varieties was collected during September 2015 in the Emilia Romagna region (Italy) and obtained by processing “Sangiovese” red wines from Caviro Extra s.p.a. (Faenza, Italy); after the collection, the pomace was immediately freeze-dried, ground with a domestic miller and stored at 4 °C until the preparation of the NaDES formulations. NaDESs (betaine: citric acid, BET-CA; betaine: ethylene glycol, BET-EG; betaine: urea, BET-U) were prepared by mixing betaine (BET; hydrogen bond acceptor (HBA)) and three different hydrogen bond donors (HBDs) at the appropriate stoichiometric ratio: citric acid (CA, 1:1), ethylene glycol (EG, 1:2), urea (U, 1:2). The mixtures were heated at 70 °C and magnetically stirred until uniform colourless liquids were obtained; then, distilled water (40 wt%) was added to get a homogeneous liquid phase. Each NaDES (10 g) was added to freeze-dried red grape pomace (250 mg) and the mixture was stirred at rt for 24 h. After 24 h, each mixture was centrifuged and separated from the residual biomass. Each extraction was performed in triplicate [4]. Since several reports have shown that the NaDES extraction method makes it possible to obtain an higher content of polyphenolics from food by-products than classical extraction with EtOH, H_2_O or a mixture of both as a solvent [4,11], in this work, grape pomace polyphenols were only extracted through selected environmentally friendly NaDES formulations.

### 2.3. HPLC-ESI-MS/MS Method

Liquid chromatography analysis was performed using a 2690 Alliance system (Waters, Milford, MA, USA). Analytical separation was performed using Phenyl-Hexyl (1.7 µm, 150 mm × 2.1 mm i.d; Waters, Milford, MA, USA). The mobile phase was constituted as follows: 15 mM ammonium acetate in water adjusted to pH 8.0 with ammonia (solvent A) and methanol 99.9% (solvent B). Chromatographic separation was achieved at a 0.15 mL min^−1^ flow rate under gradient elution conditions: 95% A for 5 min, 95–40% A from 5 to 15 min, 40–20% from 15 to 20 min, 20% A from 20 to 25 min, 20–95% A from 25 to 27 min and 95% A from 27 to 35 min. All the changes in the mobile phase composition were linear. The injected sample volume was 5 μL. The analytical column was maintained at 30 °C. The column effluent was introduced into the ESI source, operating in positive ionization mode, connected to a triple quadruple mass spectrometer (Quattro-LC, Micromass), operating in the multiple reaction monitoring (MRM) acquisition mode. Standard solutions of three anthocyanins were used to evaluate the polyphenol profiles of each extract: malvidin-3-oglucoside (MAL), quercetin-3-oglucoside (QUE) and malvidin aglycon. The most abundant signals for each compound in the MRM acquisition mode were monitored for the quantification (*m*/*z* 3309 →330.9 (malvidin, aglycon), *m*/*z* 302.9 → 302.9 (quercetin-*O*-glucoside), *m*/*z* 330.9→330.9 (malvidin-*O*-glucoside and *m*/*z* 153.6 → 125.6 (hydroxytyrosol)). The analytical method was developed and validated according to ICH guidelines to satisfy high analytical parameters in terms of accuracy and reproducibility. The limit of detection (LOD) and limit of quantification (LOQ) were determined by the signal-to-noise ratio (LOD = 3, LOQ = 10). The recovery and the matrix effect were evaluated for each compound before the sample analysis. A seven-point calibration curve (0.5, 1, 2.5, 5, 10, 25 and 50 ng mL^−1^) was used for the quantification of each compound using standard solutions in the mobile phase (phase A: phase B—95:5). An internal standard was used for calibration curves at a fixed concentration of 5 ng mL^−1^. The amount of malvidin in each formulation was checked periodically every 30 days for 2 months to determine its stability over the time.

### 2.4. Ex Vivo Permeation Study by Franz Diffusion Cells

Franz diffusion cells (Copley Scientific, Nottingham, UK) were used to perform a specific permeation study for topical formulations. This apparatus consisted of ten tailor-made donors and receptors and ten heated chambers. Mixing with a magnetic stirring bar at 300 rpm guaranteed a homogeneous temperature at 37 °C and concentration in the cell solution contents. Porcine ear skin membranes were used to perform the ex vivo permeation study. Briefly, after removing subcutaneous fat, portions of porcine ear skin were carefully cleaned with 0.9% physiological solution before storing them at −20 °C. Before each permeability experiment, tissue specimens were thawed at room temperature in PBS 0.1 M, pH = 7.4, and 5% (*m*/*V*) BSA. Thereafter, membranes were mounted in flow-through diffusion cells (exposed area: 1.8 cm^2^) with the stratum corneum facing the donator compartment and the dermis facing the receptor. The receptor compartment of the cell was filled with the medium chosen (PBS 0.1 M, pH = 7.4, and 5% *m*/*V* BSA). A quantity of 1 mL of each NaDES extract was applied to the surface of the skin/membrane in the donator compartment. Aliquots of 200 µL were collected from the receptor side at the designated time intervals (0.5, 1, 2, 4, 6 and 8 h) and immediately replaced by 200 µL of fresh medium solution. The permeated anthocyanin concentration was determined by the LC-mass spectrometry method describe above. The cumulative amount permeated per unit area was plotted versus time for the three tested NaDES extracts along with their permeability profiles at 37 °C. The steady state flux (J, μg cm^−2^ h^−1^) and lag time could be estimated by extrapolation. The malvidin amount passing through the porcine ear skin (expressed as μg/cm^2^) and the steady state flux J are reported for each tested formulation. The values reported are the means of at least five independent experiments.

### 2.5. D Keratinocyte Cell Model

An immortalized keratinocyte cell line from adult human skin (HaCaT cells), kindly gifted by Prof. F. Martini (University of Ferrara), was used for all the experiments. Cells were maintained in DMEM high glucose containing 10% FBS, 2.5 mM L-glutamine, penicillin and streptomycin at 37 °C in an atmosphere of 5% CO_2_. To obtain 3D spheroids, HaCaT cells were seeded in the “Ultra-Low Attachment Surface” 96-well black microtiter plate (Corning, Amsterdam, Netherlands), which consisted of a hydrophilic and neutrally charged hydrogel coating covalently bound with polystyrene surfaces (20,000 cells per well^−1^). This hydrogel surface naturally inhibits nonspecific interactions of the cells, eliminating unwanted cell attachment and forcing them to rest in suspension, allowing the formation of 3D spheroids. The medium culture was the same as 2D HaCaT cells and it was changed every day before experiments to obtain the spheroids’ morphology [12].

### 2.6. Quantification of Intracellular H_2_O_2_ in 3D HaCaT Cells

Three days before the experiment, HaCaT cells (20 × 10^3^ cells well^−1^) were plated in the “Ultra-Low Attachment Surface” 96-well black microtiter plate with a concave transparent bottom to allow the formation of 3D spheroids. The medium (DMEM high glucose + 10% FBS, 2.5 mM L-glutamine and antibiotic–antimycotic solution) was replaced every day, removing 50 µL and then adding the same volume for optimal cell growth. After 72 h, 50 μL of the dioxetane CL probe working solution (20 μM) was added in each well and then incubated for 20 min at 37 °C to obtain a final concentration of 5 μM/well. After this incubation, 50 μL of standard solutions of menadione were dispensed to induce intracellular H_2_O_2_ production (final concentration range 5–200 μM) and the CL signal was monitored for 40 min using a Luminoskan™ Ascent luminometric plate reader [13]. Menadione was used as an ROS-generating chemical [14]. The whole assay was performed at 37 °C and the dose–response curve was obtained by plotting the CL signal versus the concentration of the pro-oxidant and fitting the experimental data to a straight line using the method of least squares.

### 2.7. Antioxidant Activity of Red Pomace Extracts in HaCaT Spheroids

Two days before the experiment, HaCaT cells (20 × 10^3^ cells well^−1^) were plated in the “Ultra-Low Attachment Surface” 96-well black microtiter plate with a concave transparent bottom to allow the formation of 3D spheroids. The medium (DMEM high glucose + 10% FBS, 2.5 mM L-glutamine and antibiotic–antimycotic solution) was replaced every day, removing 50 µL and then adding the same volume for optimal cell growth. After 48 h, cells were treated for 24 h with serial dilutions of NaDES, with some containing polyphenolic extracts (range in content of malvidin: 0.05–1.1 μg mL^−1^). On the day of the experiment, the cell medium was collected, then 50 μL of the CL probe working solution of Aquaspark (20 μM) was added and incubated for 20 min at 37 °C to obtain a final concentration in the well of 5 μM in 150 µL. After the incubation, 50 µL of the oxidative stress inductor menadione (200 μM) was added in each well to obtain a final concentration in the well of 50 μM. CL emission signal was monitored using a Luminoskan™ Ascent luminometric plate reader. The temperature was maintained at 37 °C during the measurement [13]. 24 h treatment with malvidin in BET-CA (range of 0.07–0.6 μg mL^−1^) was used as a reference.

### 2.8. Cell Cytotoxicity: Lactate Dehydrogenase Release

Lactate dehydrogenase (LDH) release from HaCaT spheroids was monitored by collecting aliquots of the medium using a standard spectrophotometric method [14]. The method is based on a coupled enzymatic reaction in which LDH catalyses the conversion of lactate to pyruvate via NAD+ reduction to NADH. Diaphorase reduces tetrazolium salt—oxidizing NADH in the process—to a red formazan product that can be measured at 490 nm. The medium derived from HaCaT spheroids, treated for 24 h with different concentrations of NaDES extracts (range in content of malvidin: 0.05–1.1 μg mL^−1^), was collected and the increase in absorbance between the treatment after 24 h and the control was monitored at 37 °C using an Allsheng FlexA-200 Microplate Reader. Menadione (50 μM for 16 h of treatment) was used as a cytotoxic agent [14].

### 2.9. Spectrophotometric Cell Viability Bioassay

The cell viability was assessed by WST8 [2-(2-methoxy-4-nitrophenyl)-3-(4-nitrophenyl)-5-(2,4-disulfophenyl)-2*H*-tetrazolium, monosodium salt] (Dojindo Molecular Technologies, Japan), which, in the presence of an electron mediator, is reduced by dehydrogenases in cells (as a vitality biomarker) to formazan dye, which is soluble in the tissue culture medium. The amount of the formazan dye generated by dehydrogenases in cells is directly proportional to the number of living cells. The decrease in absorbance between the treatment after 24 h and the control was monitored at 37 °C at 450 nm using an Allsheng FlexA-200 Microplate Reader [15]. For this set of experiments, BET-CA formulation was added to the medium of human 3D HaCaT spheroids for 24 h (range in content of malvidin: 0.05–1.1 μg mL^−1^). The free radical-generating chemical menadione (50 μM for 16 h of treatment) was used as cytotoxic agent [14]. 24 h treatment with malvidin in BET-CA (range of 0.07–0.6 μg mL^−1^) was used as a reference.

### 2.10. Protein Release

Human HaCaT keratinocytes seeded for 48 h into the “Ultra-Low Attachment Surface” 96-well black microtiter plate with a concave transparent bottom were treated with NaDES polyphenolic extracts for 24 h and stimulated with menadione (50 μM for 16 h), as previously reported. At the end of the treatment, supernatants were collected and centrifuged to remove any cell detritus. Aliquots were stored at −80 °C until use. IL-8 and IL-l 0 protein levels were then determined using commercially available ELISA (Elabscience, US). 24 h treatment with malvidin in BET-CA (range of 0.07–0.6 μg mL^−1^) was used as a reference. The assay was performed according to the manufacturer’s instructions. Triplicate wells were used for each individual sample.

### 2.11. Statistical Analysis

Results are expressed as means ± SD of at least three independent experiments. GraphPad Prism v. 6.0 (GraphPad Software, Inc., La Jolla, CA, USA) was used to plot the experimental data and for the least-squares fitting of calibration, dose–response curves (for evaluation of IC50 values) and assay comparison graphs.

## 3. Results and Discussion

### 3.1. Extraction and Characterization of Anthocyanins in NaDES Extracts

It is well-known that skins from grape pomace are highly enriched in phenolic compounds and the main representatives of this family are anthocyanins [16]. Anthocyanins are extremely unstable and their degradation during processing is influenced by several factors, including pH [17] and light [18], so the extraction process is crucial in maintaining the stability of these compounds. The conventional extraction of polyphenols uses volatile, toxic and flammable organic solvents and usually mixtures of solvents in a multiple-step process, which can be time-consuming and expose the operator and the overall plant to different levels of risk [19]. In the present work, polyphenols from red grape pomace were extracted with the greener alternative of natural deep eutectic solvents (NaDESs), both as extracting agents and polyphenols carriers, incorporating NaDESs into bioactive formulations. NaDESs have recently been proposed as an excellent platform to enable transdermal delivery of various therapeutics, including proteins and siRNA, with a higher capacity for skin permeation and lower tendency to induce skin irritation compared to conventional chemical permeation enhancer (CPE) molecules [20].

This relatively new approach makes it possible to exploit the peculiarities of these new solvents, which are non-volatile and biocompatible, thus creating novel NaDES-based materials and at the same time improving the bioavailability, diffusion and transport of polyphenols, as well as maintaining their stability [21]. Moreover, the overall sustainability of the process is increased, thus by-passing the need for downstream purification steps aimed at NaDES removal with consequent solvent consumption. Indeed, NaDESs can be used as vehicles for anthocyanins, making it easy for the process to be scaled-up.

Three NaDESs were prepared by mixing betaine, the hydrogen bond acceptor (HBA), and three different hydrogen bond donors (HBDs), namely citric acid (BET-CA), ethylene glycol (BET-EG) and betaine-urea (BET-U). The single components were chosen based on their skin compatibility, being allowed by the European Union for cosmetic products or regularly used as active materials in cosmetic products: betaine is allowed as an antistatic/viscosity controlling agent [22], urea is allowed as an antistatic/humectant/skin conditioning agent and citric acid is allowed as a buffering/chelating agent, whereas ethylene glycol is allowed as a surfactant, emulsifier and humectant [23]. Moreover, the incorporation of these single components into NaDES mixtures has already been proven to be suitable for the extraction of polyphenols from red grape pomace: while BET-EG exhibited similar performance to hydroalcoholic mixtures, both BET-CA and BET-U had improved extraction capabilities, significantly higher than hydroalcoholic mixtures [4].

Anthocyanin content in the three formulations was determined by HPLC-ESI MS/MS. Analytical calibration curve parameters for quantification of the NaDES extracts, in the range 0.5–100 ng mL^−1^, were obtained from the plot of the analyte/internal standard peak area ratio versus the analyte concentration using a linear least-square regression analysis. The obtained analytical calibration curve equations were expressed in the form of *Y* = (a ± δa) X + (b ± δb). Determination coefficients (*r*^2^) of the analytical calibration curves were ≥0.990 for all analytes (see the Supplementary Materials). Limit of detection values (LOD) were 0.1 ng mL^−1^, while limit of quantification values (LOQ) were 0.5 ng mL^−1^ for all analytes. Variation coefficients and bias percentage, calculated intra- and inter-daily, were less than 5% for all analytes at the three different concentration levels, indicating the appropriate precision and accuracy of the developed method. The evaluation between standard solutions and fortified samples with known amounts of analyte and blank samples showed good selectivity in multiple reaction monitoring (MRM) mode, as no interfering signals potentially able to affect the identification of the target analytes were observed. The matrix effect percentage was evaluated at three concentration levels in all the studied matrices. In all the studies, matrix effect values were negligible, always being below 10% [24].

The results showed that malvidin was the major anthocyanin identified, with a similar content in all the formulations: 56.66 μg mL^−1^ for BET-CA, 52.41 μg mL^−1^ for BET-U and 51.69 μg mL^−1^ for BET-EG. Malvidin-3-*O*-glucoside and quercetin-3-*O*-glucoside were not detected in the analysed NaDES extracts.

### 3.2. Skin Permeation of Anthocyanins Derived from NaDES Extracts

Anthocyanins are increasing in popularity in the food industry but have been poorly investigated as bioactive molecules in topical formulations. In order to exert their biological effects, they must be released from the developed topical formulations on the skin surface, overcome the barrier function of the stratum corneum (SC) and penetrate into the epidermis and dermis [25]. From the therapeutic point of view, the bioavailability is a crucial parameter allowing their biological effects [26]. Anthocyanins constitute the largest group of water-soluble molecules in plants but have limited bioavailability in skin as they are poor lipophilic compounds. Their skin penetration can be enhanced by a proper formulation that constitutes a complex of the excipients and vehicles used, allowing the partition in the lipid domain of the skin structure [27]. NaDESs show promise as drug carriers in dermal formulations or formulations for local administration in the oral cavity due to their low toxicity, tunability and biodegradability, as well as their solubilizing and stabilizing properties [28].

Since the three NaDES formulations showed similar amounts of malvidin aglycone, we directly focused on the bioavailability using Franz cells as an ex vivo permeation model.

As can be seen in the Table 1, the BET-CA formulation seemed to be the one that had major malvidin permeability through the membrane; therefore, we decided to further analyse only this formulation as the best possible excipient in a cosmetic product for topical use.

**Figure 1:**
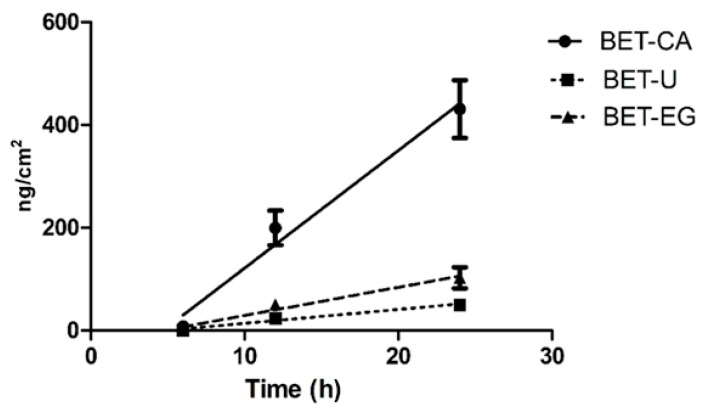
Permeation profiles of the three NaDES formulations (24 h experiment). The cumulative amount permeated per unit area was plotted versus time for the three tested NaDES extracts and their permeability profiles at 37 °C. The malvidin amounts passed through the porcine ear skin (expressed as μg/cm^2^) and the steady state flux J are reported for each tested formulation at the selected temperature. The values reported are the means of at least five independent experiments.

In Figure 1, a graph with the malvidin amounts permeated (ng cm^−2^) versus time is shown. The difference between BET-CA and the other formulations was significant (*p* < 0.01). Since anthocyanins are more stable at low pH (acidic conditions) [29], we hypothesized that the BET-CA formulation preserved the stability of this molecule.

### 3.3. Safety of NaDES Extracts In Vitro in Human 3D Keratinocytes

**Figure 2:**
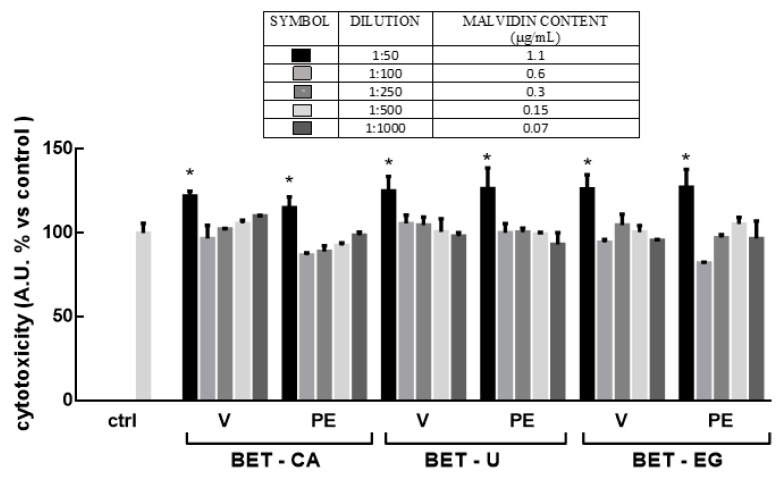
HaCat spheroids were treated with pomace extracts (PE) in NaDES formulations (range in content of malvidin: 0.05–1.1 μg mL^−1^) for 24 h: betaine—citric acid (BET-CA), betaine—urea (BET-U) and betaine—ethylen glycol (BET-EG). Corresponding dilutions of the NaDES mixtures used for extraction, without grape pomace extracts, were utilized as the control (vehicle (V)). LDH activity was spectrophotometrically quantified in a cellular medium as an index of cytotoxicity, as described in the Materials and Methods section. Results are expressed as means ± SD of three independent experiments, each performed in triplicate. * *p* ˂ 0.001 significantly different from the control (ctrl).

Cell-based assays are one of the most appealing tools for the evaluation of a plethora of biological activities in the cosmetic area, since they allow highly human and predictive information that makes it possible to avoid or reduce in vivo experiments in compliance with the guiding principle of the “Three Rs” (replacement, reduction and refinement) concerning the use of animals in scientific research. 3D cell culture systems overcome many of the limitations of conventional 2D cells, more closely mimicking the complex phenotypic heterogeneity that chemical gradients produce during cell growth [10]. In this study, we used low-attachment-based generation of human HaCaT spheroids, as reported by Klicks et al. [10], to evaluate the suitable and safe concentrations of NaDES formulations and their biological activities. Firstly, the safety of all the formulations was investigated by quantifying the release of lactate dehydrogenase (LDH), a marker for cell death both in vitro and in vivo. HaCaT spheroids were treated with different dilutions of the three formulations (range: 1:50–1:1000 *v*/*v*, corresponding to 1.1 ± 0.1 μg mL^−1^ of malvidin), and LDH was quantified in cell culture medium. As shown in Figure 2, all the formulations tested at the dilution 1:50 *v/v* significantly increased LDH release (*p* ˂ 0.001), while lower dilutions were not cytotoxic towards the cells and thus considered safe. These data are in line with previous results obtained in 2D HaCat [30], showing good biocompatibility for the tested cells.

Previously, results obtained by HPLC/ESI MS-MS analysis showed similar malvidin contents in the three formulations, while malvidin in the BET-CA formulation showed the highest bioavailability in the ex vivo permeation studies. We thus further analysed this formulation to determine its antioxidant and anti-inflammatory effects in human 3D keratinocytes, in a range of dilutions related to malvidin contents between 0.6 and 0.07 μg mL^−1^.

### 3.4. Evaluation of Intracellular H_2_O_2_ Production in Human 3D Keratinocytes

The cell-based bioassay recently developed by us to quantify the intracellular production of H_2_O_2_ in human living cells [13] was employed in 3D keratinocytes cultures upon treatment with the pro-oxidant agent menadione (2-metil-1,4-naftochinone), also known as vitamin K3 [14]. The selectivity against H_2_O_2_ is due to the salicylaldehyde (the sensing moiety) in the aromatic ring of the aryl-adamantylidene−dioxetane CL probe [31]; indeed, upon reaction with H_2_O_2_, the salicylaldehyde is oxidized to a catechol, followed by a subsequent ether cleavage to unmask the phenoxy-dioxetane, which further undergoes chemiexcitation to release the chemiluminescent signal at 540 nm [31]. Moreover, the presence of an electron-withdrawing acrylic group in the aromatic ring results in the production of a singlet excited state species that emits light with greater efficiency in aqueous solution, thus increasing CL emission intensity without the use of additional enhancers, allowing the probe to behave as an all-in-one reagent. Finally, the CL probe is able to permeate inside cells without affecting cell viability, thus making t possible to perform experiments in living cells without using any lysing agents [13].

**Figure 3:**
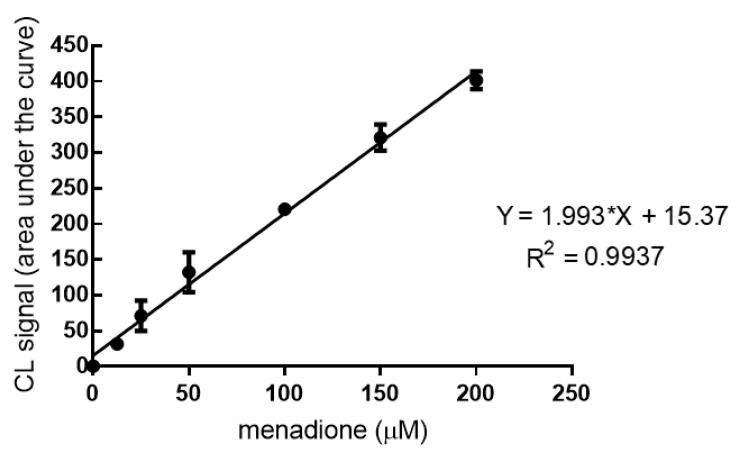
Chemiluminescence kinetic profiles obtained for human HaCat spheroids in the presence of the dioxetane CL probe and different concentrations of menadione (range: 5–200 μM).

Human HaCat spheroids in the wells of the “Ultra-Low Attachment Surface” 96-well black microtiter plate were incubated for 20 min with the CL probe working solution (5 µM of CL probe in 0.1 M PBS pH 7.4) and then treated with menadione (range 5–200 μM) to measure the intracellular production of H_2_O_2_ upon the pro-oxidant stimulus. The CL signal of the reaction was monitored for 40 min using a Luminoskan™ Ascent luminometric plate reader. As expected, the CL emission intensity increased with the concentration of the stimulus (Figure 3).

A good correlation between the CL signal and the concentration of menadione was observed, which reflects the physiological mechanisms of intracellular H_2_O_2_ production. The concentrations of the pro-oxidant agent menadione required to obtain a detectable increase in the CL signal were in the micromolar range (LOD and LOQ were 6.3 µM and 8.0 µM, respectively).

### 3.5. Antioxidant Activity of NaDES Extracts toward Human HaCaT Spheroids

This CL effect-based bioassay was utilized to evaluate the antioxidant activity of the BET-CA pomace extracts toward human HaCaT spheroids, as this was the formulation that showed more efficient permeation in skin.

Oxidative stress is thought to play a central role in initiating and driving the signalling events that lead to cellular response and damage following UV irradiation. Indeed, ROS generated in keratinocytes are rapidly removed by non-enzymatic and enzymatic antioxidant substances to protect the living system from their harmful effects. These factors maintain a pro-oxidant/antioxidant balance, resulting in the stabilization of cell structure. An excess of free radicals results in a cascade of events mediating progressive deterioration of cellular structure and function, leading to a loss of cellular integrity by modification of DNA and also to abnormal expression of cellular genes [32]. To test the antioxidant efficacy of the BET-CA formulation, HaCaT spheroids were treated for 24 h with increased concentrations of BET-CA, in the range of 0.07–0.6 μg mL^−1^ of malvidin, and then injured with menadione.

Specifically, on the day of the experiment, the cell medium containing BET-CA was changed to a fresh one and cells were challenged with the pro-oxidant menadione (25 μM). The CL signal decreased proportionally to the concentration of malvidin present in the formulation, obtaining an IC50 value of 0.15 ± 0.02 μg mL^−1^. We performed, in parallel, the same experiment but treated cells for 24 h with malvidin in a BET-CA vehicle (range: 0.07–0.6 μg mL^−1^), obtaining an IC50 of 0.20 ± 0.04 μg mL^−1^. These results suggest that the BET-CA formulation can reduce intracellular H_2_O_2_ production at least in part thanks to malvidin-derived modulation of intracellular pro-oxidant or antioxidant factors.

### 3.6. Protective Effects of NaDES Extracts on HaCat Spheroids in the Presence of the Cytotoxic Agent Menadione

HaCat spheroids were used to preserve the functionality and responses of human keratinocytes towards the BET-CA formulation since inflammatory responses were initiated in the outer boundary of the skin. The release of pro-inflammatory cytokines and chemokines is one of the adaptive immune responses towards inflammatory stimuli. ROS production derived from exposure to environmental and toxic chemicals, along with UV irradiation, induce keratinocytes to release a wide array of mediators, such as interleukin (IL)-8, -6, -1β and tumor necrosis factor α (TNFα) [33,34]. Several studies have suggested that nuclear factor kappaB (NF-kB) and activator protein 1 (AP-1) are redox-regulated transcription factors and that their activation by different agents, including UV light exposure and pro-oxidant factors, is a determinant in promoting the expression of genes related to the inflammatory pathway. Among them, IL-8 plays a pivotal role in cutaneous inflammation and has been implicated in tumour promotion [34].

**Figure 4:**
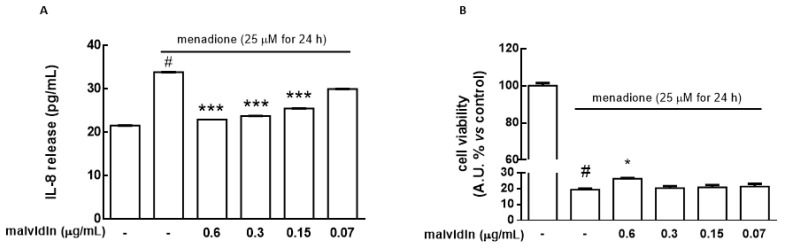
HaCat spheroids were treated with several dilutions of the BET-CA formulation (range of malvidin: 0.07–0.6 μg mL^−1^) for 24 h, including 16 h in the presence of menadione (25 μM). (**A**) Release of the proinflammatory cytokine IL-8 (pg mL^−1^) was determined by ELISA assay. (**B**) Cell viability was measured by cell counting kit 8, as described in the Materials and Methods section. Untreated HaCat spheroids served as a control (ctrl). Results are expressed as means ± SD of three independent experiments, each performed in triplicate. * *p* ˂ 0.05, *** *p* ˂ 0.001 significantly different from menadione treatment; # *p* ˂ 0.001 significantly different from the ctrl.

Figure 4A shows that IL-8 release significantly increased (*p* ˂ 0.001) in HaCat spheroids challenged with menadione (25 μM for 16 h) while pre-treatment with the BET-CA formulation (range of malvidin: 0.15–0.6 μg mL^−1^) partially counteracted IL-8 production (*p* ˂ 0.001).

Finally, we evaluated the ability of the BET-CA formulation to counteract the cytotoxicity induced by menadione in a concentration range in which it was able to exert its antioxidant activity toward HaCat spheroids. Cells were treated for 24 h with BET-CA (malvidin content range: 0.6–0.15 μg mL^−1^), including 16 h in the presence of menadione (25 μM). The results showed that menadione treatment (25 μM for 16 h) significantly decreased the cell viability of HaCat spheroids (*p* ˂ 0.001) and that BET-CA (malvidin content: 0.6 μg mL^−1^) was able to significantly counteract the activity of the cytotoxic agent (*p* ˂ 0.05), suggesting that pomace extract exerts a beneficial, preventive role in keratinocytes against harmful agents (Figure 4B).

To better understand if the malvidin in the BET-CA formulation was primarily responsible for the protective effects in IL-8 release and cell viability in HaCat spheroids, we performed, in parallel, experiments treating cells for 24 h with malvidin with the BET-CA vehicle (range 0.07–0.6 μg mL^−1^), including 16 h in the presence of menadione (25 μM). Supplementary Figures S1 and S2 show that malvidin with the BET-CA treatment was able to significantly counteract IL-8 release and the cytotoxic activity of menadione.

## 4. Conclusions

The sustainable exploitation of grape pomace is a useful strategy for wineries to reduce environmental contamination and as an alternative to reduce the carbon footprint in the whole production process. Its major use is in biogas or compost production, but more valuable routes can be explored. Here, we report an integrated/combined valorisation of grape pomace that involved NaDES extraction and bioanalytical analysis to characterize the pomace’s content and bioavailability, through HPLC-ESI MS/MS and biological activities, along with the use of smart, highly predictive and effect-based intracellular bioassays in 3D human keratinocytes, for cosmeceutical applications. In this work, NaDES efficiently replaced organic solvents and fulfilled the requirements of the sustainable development concept while maintaining the high-quality standards of safety and beneficial effects that cosmetic products need. In addition, the same cocktail for extraction was directly used as a safe vehicle for the topical formulation proposed. This innovative process can be easily scaled-up to obtain an efficient and sustainable method that can be applied not only for cosmetic purpose but also for oral and genital mucosa applications and in veterinary medicine.

The characterization of grape pomace extracts in NaDES formulations and the bioavailability of their bioactive molecules are relevant and key points to increase the economic value of the obtained product. The BET-CA formulation not only exhibited the highest malvidin bioavailability but also good antioxidant and anti-inflammatory effects at concentrations able to permeate in skin.

Studies on the identities and individual concentrations of phenolics recovered after extraction, as well as their biological effects, support the application of extracts in topical preparations [35,36]. We developed a simplified and eco-sustainable process with the aim of making production easier to scale-up, as well cheaper, thus facilitating the valorisation of food waste. Moreover, our findings also support the use of NADES as promising carriers in new drug delivery systems for topical applications, since they can affect the permeation of active molecules.

In conclusion, the waste matrices of the wine production chain can represent a promising starting material for the acquisition of active ingredients to be included in topically applied cosmetic products, as they are able to reduce excessive oxidative stress and inflammatory processes, thus preventing cellular aging.

## Figures and Tables

**Table 1:** Amounts of malvidin permeated (ng cm^−2^) for the three tested formulations at 37 °C.

Time (Hours)	BET-CA (ng cm^−2^) ± SD	BET-U (ng cm^−2^) ± SD	BET-EG (ng cm^−2^) ± SD
6	8.35 ± 4.71	0.00	0.00
12	213 ± 43	28.1 ± 2.5	49.7 ± 10.2
24	431 ± 7	50.4 ± 14.9	103 ± 5
Flow J (ng cm^−2^ h^−1^)	23.4 ± 3.1	2.80 ± 0.48	5.71 ± 1.14

## Data Availability

Data presented in this study are available on reasonable request.

## Supplementary Materials

The following are available online at https://www.mdpi.com/article/10.3390/biom11081181/s1, Figure S1: HaCat spheroids were treated for 24 h with malvidin (range 0.07–0.6 μg mL^−1^) dissolved in BET-CA vehicle, in the presence of menadione (25 μM) for 16 h.
