# Supplementary material for: Grape Pomace for Topical Application: Green NaDES Sustainable Extraction, Skin Permeation Studies, Antioxidant and Anti-Inflammatory Activities Characterization in 3D Human Keratinocytes
Source: Biomolecules. 2021 Aug 10;11(8):1181. doi: 10.3390/biom11081181 (PMC8393215; doi:10.3390/biom11081181)
Supplement: Supplementary file 1 [file biomolecules-11-01181-s001.zip › biomolecules-1312051-supplementary.pdf]

Supplementary Material

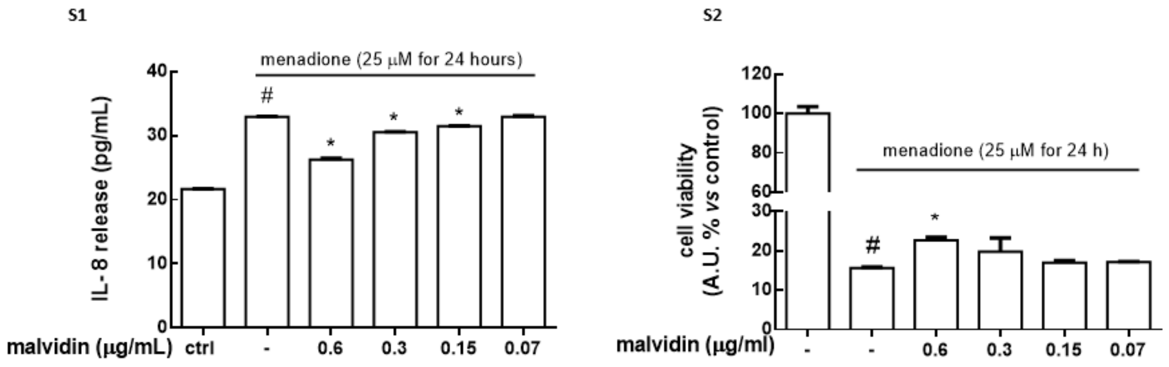

Supplementary Figures. HaCat spheroids were treated for 24 h with malvidin (range 0.07 – 0.6 μg mL−1) dissolved in BET-CA vehicle, in the presence of menadione (25 μM) for 16 h. 1) Release of the proinflammatory cytokine IL-8 (pg mL−1) was determined by ELISA assay. 2) Cell viability was measured by cell counting kit-8 as described in Materials and Methods Section. HaCat spheroids not treated served as a control (ctrl). Results are expressed as mean ± SD of three independent experiments, each performed in triplicate. *P<0.05, ***P<0.001 significantly different from menadione treatment; # P<0.001 significantly different from the ctrl.
